# Correlation of Lipid Ratios With the Severity of Pulmonary Alveolar Proteinosis: A Cross-Sectional Study

**DOI:** 10.3389/fnut.2021.610765

**Published:** 2021-03-18

**Authors:** Xin Yan, Yujuan Gao, Qi Zhao, Xiaohua Qiu, Mi Tian, Jinghong Dai, Yi Zhuang

**Affiliations:** Department of Respiratory and Critical Care Medicine, Nanjing Drum Tower Hospital, The Affiliated Hospital of Nanjing University Medical School, Nanjing, China

**Keywords:** lipid ratio, pulmonary alveolar proteinosis, severity, TC/HDL-C ratio, TG/HDL-C

## Abstract

**Background:** Lipids are known to accumulate abnormally in the alveoli and circulate during pulmonary alveolar proteinosis (PAP). However, the relationship between lipid ratios and PAP is not clear. In this study, we investigated the lipid ratios in PAP patients and explored the relationships between lipid ratios and the severity of PAP.

**Methods:** A total of 122 PAP patients were diagnosed and divided the mild- moderate PAP group (*n* = 61) and the severe PAP group (*n* = 61) according to the value of disease severity score (DSS). One hundred thirty healthy volunteers were classified as the control group. Routine blood examination and pulmonary function tests were performed and lipid profile were measured.

**Results:** Compared with the control group, patients with PAP had significantly higher TG, TC/HDL-C, TG/HDL-C, and non-HDL-C, while lower HDL-C (all *P* < 0.05). Patients with the severe PAP had higher TC, TG, LDL-C, TC/HDL-C, and non-HDL-C, while lower HDL-C than patients with the mild- moderate PAP (all *P* < 0.05). Binary logistic regression analysis indicated that TC/HDL-C (*OR* = 2.322, 95% CI 1.621–3.713, *P* = 0.024) and non-HDL-C (*OR* = 1.797, 95% CI 1.239–3.109, *P* = 0.036) were all significantly correlated with the severity of PAP after adjustment for other risk factors. The AUC value of TC/HDL-C for predicting the severity of PAP was larger than that of non-HDL-C. The AUROC for TC/HDL-C was 0.741 (0.654–0.828), and the optimal cut-off point for TC/HDL-C was 5.05 (sensitivity: 73.6%, specificity: 68.1%).

**Conclusions:** Lipid ratios, including TC-HDL-C and non-HDL-C, were independent risk factors for the severity of PAP. TC/HDL-C is a promising biomarker for the severity of PAP.

## Introduction

Pulmonary alveolar proteinosis (PAP) is a rare and serious condition in which excessive lipids and surfactant proteins build up in alveolar macrophages and alveoli, resulting in impaired gas exchange (1, 2). PAP has a diverse clinical course, ranging from spontaneous remission to death from progressive respiratory failure (3). The severity of PAP is usually based on pulmonary function test results such as reduction of the diffusing capacity of the lungs for carbon monoxide (DL_CO_) and gas transfer [e.g., partial pressure of arterial oxygen [PaO2], Alveolar-arterial Oxygen Gradient (A-aDO2) (4, 5)]. However, the pulmonary function test requires high operational demands on the part of the doctor and close cooperation between the patient, and blood gas analyses was invasive. Thus, simple, inexpensive, and readily accessible biomarkers of the severity of PAP represent an important advance for this purpose.

In the past few years, the lipid ratios, such as total cholesterol/high-density lipoprotein cholesterol (TC/HDL-C) and triglyceride/high-density lipoprotein cholesterol (TG/HDL-C) have been proposed as alternative biomarkers for predicting Type 2 diabetes (T2DM), cardiovascular disease, and metabolic syndrome (6, 7). In addition, the relationship between lipid ratios and pulmonary disease has received increasing attention, with a positive association between TG/HDL-C and pulmonary disease reported in asthma and obstructive sleep apnea (8, 9). An increased TG/HDL-C ratio is a marker for systemic inflammation in patients with idiopathic pulmonary arterial hypertension (10). Numerous studies have confirmed that patients with PAP have elevated pulmonary and circulating lipoproteins (11, 12). However, there are limited data available about the role of lipid ratios in PAP.

Therefore, in this study, we explored the association between lipid ratios and PAP. In addition, this study compared the predictive significance for the severity of PAP between lipid ratios and traditional lipid indicators, such as TC, low-density lipoprotein cholesterol (LDL-C), TG, and HDL-C.

## Participants and Methods

### Study Population

This study included 122 PAP patients who were recruited from the inpatient of the Department of Respiration of Nanjing Drum Tower Hospital between January 2010 and June 2018 and diagnosed by transbronchial lung biopsy. All 122 patients were received anti-Granulocyte–macrophage colony-stimulating factor (GM-CSF) autoantibody testing, 6 of them were diagnosed as non-autoimmune PAP. One hundred thirty healthy adults who had no medical histories were randomly selected following examination in the clinic, and were classified as the control group. All subjects gave informed written consent to participate.

Exclusion criteria for all participants were: (1) subjects had a history of sarcoidosis, occupational lung disease, idiopathic pulmonary fibrosis, pulmonary tuberculosis, chronic obstructive pulmonary disease (COPD) and cancer; (2) subjects had a history of diabetes mellitus, cardiovascular and cerebrovascular diseases, chronic liver or kidney disease, infection; and (3) subjects taking corticosteroids or lipid-lowering agents.

### Method

#### Laboratory Tests

The white blood cell count (WBCC) and neutrophil count were determined using an automated blood cell counter (Beckman Coulter Ireland Inc., Mervue, Galway, Ireland).

Fasting blood samples were collected after at least 10 h overnight and analyzed for the biochemical measurements. Routine biochemical analyses including plasma lipids and serum lactate dehydrogenase (LDH) were measured with commercial kits using an automated chemistry analyzer (Chemistry Analyzer Au2700, Olympus Medical Engineering Company, Japan). Carcinoembryonic antigen (CEA) and CYFRA21-1 were measured with an enzyme immunoradiometric assay kit (TFB, Tokyo, Japan). Blood gas analyses and pulmonary function tests including forced expiratory volume in 1 s (FEV1), forced vital capacity (FVC), and carbon monoxide diffusion capacity (DLco) were performed in PAP patients.

#### Assessment of Disease Severity Score

Each patient was assigned a PAP disease severity score (DSS) based on the presence/absence of symptoms and the degree of PaO2 at initial diagnosis, as previously described (5). The categories of score ranged from DSS 1 to DSS 5: DSS 1 = no symptoms and PaO2 ≥70 mmHg, DSS 2 = symptomatic and PaO2 ≥70 mmHg, DSS 3 = 60 mmHg ≤ PaO2 <70 mmHg, DSS 4 = 50 mmHg ≤ PaO2 <60 mmHg, DSS 5 = PaO2 <50 mmHg. PAP patients were further divided into two groups according to DSS): the mild- moderate PAP group (patients with low DSS [DSS 1–2], *n* = 61) and the severe PAP group (patients with high DSS [DSS 3–5], *n* = 61).

#### Statistical Analysis

Continuous variables were expressed as mean ± SD. Differences between two groups were tested by *t*-test. Categorical variables were expressed as percentages (numbers) and analyzed by Pearson's Chi-squared test. The binary logistic regression analysis was performed to investigate the independent predictors of the occurrence and severity of PAP. Receiver operator characteristic (ROC) analyses were performed to calculate area under the ROC curve (AUC) of each lipid parameter for the severity of PAP. Data were analyzed using SPSS18.0 statistical software, with significance defined as *p* < 0.05 (two-sided).

## Results

### Baseline Characteristics of the Study Population

The patient characteristics of the PAP group were compared with those of the healthy control group, as shown in Table 1. Compared to the control group, patients with PAP had higher WBCC neutrophil count, TG, TC/HDL-C, TG/HDL-C, non-HDL-C, LDL-C/HDL-C, LDH, CYFRA21-1, and CEA (all *P* < 0.05). HDL-C was lower in patients with PAP than that in the control group. However, there were no differences of age, gender, smoking status, SBP, DBP, TC, and LDL-C between the two groups.

**Table 1:** Demographic and clinical characteristics of the patients and healthy subjects.

	**Control (*n* = 130)**	**PAP patients (*n* = 122)**	***P-*value**
Age (years)	46.93 ± 10.88	46.61 ± 11.08	0.22
Sex (male/Female)	83/47	77/45	0.24
Smoking (yes/no)	75/55	72/50	0.31
SBP (mmHg)	122.85 ± 17.69	126.28 ± 10.94	0.35
DBP (mmHg)	74.67 ± 11.15	76.69 ± 7.72	0.42
WBCC (10^9^/L)	5.16 ± 1.41	6.45 ± 1.56	<0.01
Neutrophil count (10^9^/L)	2.97 ± 1.15	3.88 ± 1.14	<0.01
TC (mmol/L)	4.76 ± 0.71	4.83 ± 0.92	0.54
TG (mmol/L)	0.76 ± 0.17	1.65 ± 0.94	<0.01
LDL-C (mmol/L)	2.69 ± 0.59	2.78 ± 0.79	0.29
HDL-C (mmol/L)	1.70 ± 0.31	1.07 ± 0.39	<0.01
TG/HDL-C	0.46 ± 0.15	1.81 ± 1.44	<0.01
TC/HDL-C	2.87 ± 0.52	5.23 ± 1.42	<0.01
Non-HDL-C	3.07 ± 0.65	3.76 ± 1.07	<0.01
LDL-C/HDL-C	1.63 ± 0.45	3.07 ± 1.69	<0.01
LDH (IU/L)	172.99 ± 18.11	284.52 ± 21.95	<0.01
CYFRA21-1 (ng/ml)	2.64 ± 0.18	10.18 ± 2.23	<0.01
CEA (ng/ml)	2.01 ± 0.11	4.68 ± 0.14	0.02
FVC pred (%)	–	84.03 ± 18.30	–
FEV1 pred (%)	–	87.05 ± 18.83	–
DL_CO_ (%)	–	63.72 ± 15.82	–

*SBP, systolic blood pressure; DBP, diastolic blood pressure; WBCC, white blood cell count; TC, total cholesterol; TG, triglyceride; LDL-C, low-density lipoprotein cholesterol; HDL-C, high-density lipoprotein cholesterol; LDH, lactic dehydrogenase; CEA, carcinoembryonic antigen; FVC, forced vital capacity; FEV1, forced expiratory volume in 1 s; DLco, carbon monoxide diffusion capacity*.

### Lipid Ratios Are Associated With the Severity of PAP

Patients with PAP were further divided into two groups according to the value of disease severity score (DSS): the mild- moderate PAP group (*n* = 61) and the severe PAP group (*n* = 61). Patients with the severe PAP had higher TC, TG, LDL-C, TC/HDL-C, non-HDL-C, and LDL-C/HDL-C than patients with the mild- moderate PAP (all *P* < 0.05). HDL-C was lower in patients with severe PAP than that in the mild- moderate group. There was no difference of TG/HDL-C between the two groups (Table 2).

**Table 2:** The differences of lipid ratios in different severity PAP groups.

	**The mild-moderate PAP group (*n* = 61)**	**The severe PAP group (*n* = 61)**	***P-*value**
Age (years)	47.72 ± 11.02	45.17 ± 11.10	0.21
Sex (male/Female)	40/21	37/24	0.33
Smoking (yes/no)	38/23	39/22	0.29
TC (mmol/L)	4.62 ± 0.95	5.10 ± 0.82	0.01
TG (mmol/L)	1.19 ± 0.56	2.25 ± 1.00	<0.01
LDL-C (mmol/L)	2.61 ± 0.78	3.01 ± 0.77	0.01
HDL-C (mmol/L)	1.19 ± 0.41	0.91 ± 0.31	<0.01
TG/HDL-C	1.65 ± 1.42	2.03 ± 1.44	0.14
TC/HDL-C	4.41 ± 2.02	6.29 ± 2.49	<0.01
Non-HDL-C	3.43 ± 1.09	4.19 ± 0.89	<0.01
LDL-C/HDL-C	2.50 ± 1.27	3.82 ± 1.88	<0.01
LDH (IU/L)	222.65 ± 18.63	365.06 ± 29.21	<0.01
CYFRA21-1 (ng/ml)	6.47 ± 4.96	15.02 ± 6.67	<0.01
CEA (ng/ml)	3.24 ± 1.21	6.55 ± 2.07	<0.01
FVC pred (%)	93.17 ± 13.21	72.31 ± 17.29	<0.01
FEV1 pred (%)	96.30 ± 13.48	75.17 ± 18.12	<0.01
DL_CO_ (%)	81.86 ± 14.72	40.44 ± 16.62	<0.01

### Binary Logistic Regression Analysis for the Risk of the Occurrence of PAP

Binary logistic regression analyses were used to identify the relationship between the lipid ratios and the occurrence of PAP. After adjusting for age, sex, smoking, SBP, DBP, WBCC, neutrophil count, LDH, CYFRA21-1, and CEA, TG (*OR* = 3.323, 95% CI 2.741–4.442, *P* < 0.001), HDL-C (*OR* = 0.417, 95% CI 0.205–0.659, *P* = 0.005), TC/HDL-C (*OR* = 3.623, 95% CI 2.189–5.997, *P* < 0.001), TG/HDL-C (*OR* = 2.751, 95% CI 1.469–3.292, *P* < 0.001), non-HDL-C (*OR* = 1.736, 95% CI 1.174–2.567, *P* = 0.005), and LDL-C/HDL-C (*OR* = 3.213, 95% CI 2.014–5.770, *P* < 0.001) were all significantly correlated with the occurrence of PAP (Table 3).

**Table 3:** Logistic regression analysis of the association between PAP and lipid ratios.

	**Model**	***OR***	**95% *CI***	***P***
TG (mmol/L)	1	3.685	2.601–5.124	<0.001
	2	3.323	2.741–4.442	<0.001
HDLC-(mmol/L)	1	0.312	0.214–0.506	<0.001
	2	0.417	0.205–0.659	0.005
TC/HDL-C	1	4.706	3.031–6.307	<0.001
	2	3.623	2.189–5.997	<0.001
TG/HDL-C	1	3.115	2.046–5.004	<0.001
	2	2.751	1.469–3.292	<0.001
Non-HDL-C	1	2.430	1.765–3.345	<0.001
	2	1.736	1.174–2.567	0.006
LDL-C/HDL-C	1	4.352	2.970–6.005	<0.001
	2	3.213	2.014–5.770	<0.001

*Model 1: unadjusted*.

*Model 2: adjustment for age, sex, smoking, SBP, DBP, WBCC, neutrophil count, LDH, CYFRA21-1, and CEA*.

### Binary Logistic Regression Analysis for the Risk of the Severity of PAP

Binary logistic regression analyses were used to identify the relationship between the lipid ratios and the severity of PAP. After adjusting for age, sex, smoking, SBP, DBP, WBCC, neutrophil count, LDH, CYFRA21-1, CEA, FVC pred, FEV1 pred, and DLCO-SB, TG (*OR* = 2.113, 95% CI 1.517–3.316, *P* < 0.001), HDL-C (*OR* = 0.217, 95% CI 0.133–0.407, *P* = 0.038), TC/HDL-C (*OR* = 2.322, 95% CI 1.621–3.713, *P* = 0.024), and non-HDL-C (*OR* = 1.797, 95% CI 1.239–3.109, *P* = 0.036) were all significantly correlated with the severity of PAP (Table 4).

**Table 4:** Logistic regression analysis of the association between the severity of PAP and lipid ratios.

	**Mode1**	***OR***	**95% *CI***	***P***
TC (mmol/L)	1	1.831	1.198–2.799	0.005
	2	1.617	0.894–2.927	0.112
TG (mmol/L)	1	2.102	1.564–3.432	<0.001
	2	2.113	1.517–3.316	<0.001
LDL-C (mmol/L)	1	1.970	1.215–3.195	0.011
	2	1.472	0.716–2.027	0.293
HDL-C (mmol/L)	1	0.305	0.232–0.445	<0.001
	2	0.217	0.133–0.407	0.038
TC/HDL-C	1	2.457	2.205–3.762	<0.001
	2	2.322	1.621–3.713	0.024
Non-HDL-C	1	2.118	1.424–3.150	<0.001
	2	1.797	1.239–3.109	0.036
LDL-C/HDL-C	1	1.720	1.310–2.258	<0.001
	2	1.663	1.160–2.383	0.006

*Model 1: unadjusted*.

*Model 2: adjustment for age, sex, smoking, SBP, DBP, WBCC, neutrophil count, LDH, CYFRA21-1, CEA, FVC pred, FEV1 pred, and DLCO-SB*.

### Diagnostic Value of Lipid Parameters for the Severity of PAP

**Figure 1:**
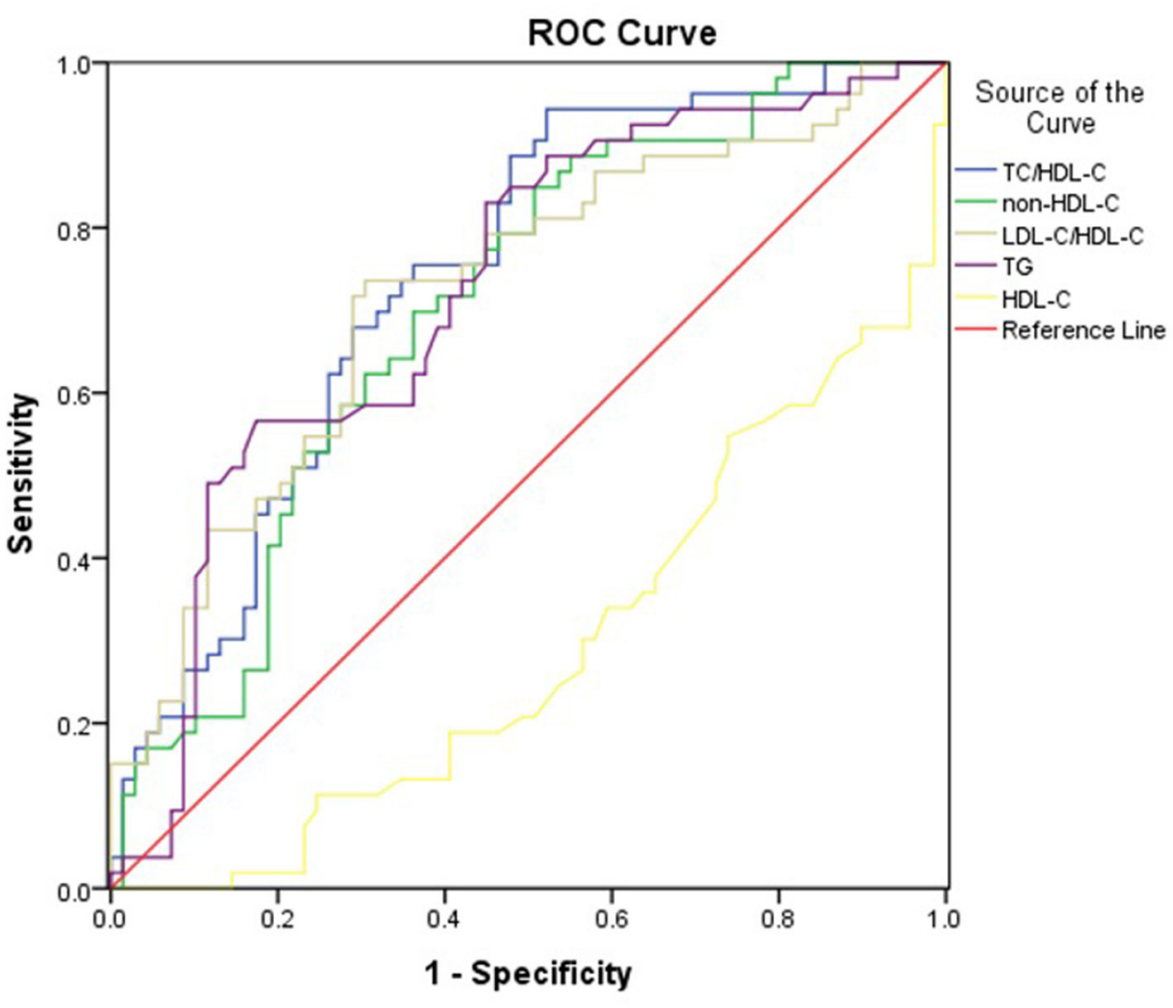
Area under the receiver operating characteristics curves (AUROCs) of lipid markers for the severity of PAP.

Table 5 and Figure 1 showed the cut-off points of lipid parameters for the prediction of the severity of PAP with their corresponding specificity and sensitivity. The AUC value of TC/HDL-C was larger than that of TG, HDL-C, and non-HDL-C. The AUROC for TC/HDL-C was 0.741 (0.654–0.828), and the optimal cut-off point for TC/HDL-C was 5.05 (sensitivity: 73.6%, specificity: 68.1%). The results indicated that TC/HDL-C had better predictive effects than other lipid parameters and was an acceptable predictor of the severity of PAP (Table 5).

**Table 5:** ROC curve for predicting the severity of PAP and cutoff points for maximum sum of sensitivity and specificity.

	**ROC (95% *CI*)**	***P***	**Cutoff point**	**Sensitivity (%)**	**Specificity (%)**
TG (mmol/L)	0.724 (0.635–0.764)	<0.001	1.69	69.8	61.2
HDL-C (mmol/L)	0.292 (0.201–0.384)	<0.001	1.01	65.2	69.6
TC/HDL-C	0.741 (0.654–0.828)	<0.001	5.05	73.6	68.1
Non-HDL-C	0.703 (0.611–0.795)	<0.001	3.96	64.2	66.6
LDL-C/HDL-C	0.722 (0.630–0.814)	<0.001	2.73	71.7	67.0

## Discussion

In this study, we demonstrated that patients with PAP had higher lipid ratios, including TC/HDL-C, TG/HDL-C, and non-HDL-C than those in the healthy control group. Importantly, we also found that TC/HDL-C was positively associated with the diagnosis and severity of PAP. Moreover, TC/HDL-C was observed to be a better predictor of the severity of PAP compared with other lipid parameters.

Lung surfactant consists of 80% polar lipids, mainly phosphatidylcholine, and several less common phospholipid species, 10% neutral lipids, mainly free cholesterol with small amounts of triglycerides and free fatty acids, and 10% surfactant protein (13). Surfactant homeostasis is maintained by secretion and recirculation of type II alveolar epithelial cells and catabolism of alveolar macrophages (14). PAP, a known disorder of surfactant homeostasis, is associated with the accumulation of surfactant lipids and proteins in the airways and alveoli that impair gas-blood exchange, shortness of breath, fatigue, and exercise intolerance. At least 80% of the cholesterol present in the lungs and almost all of the cholesterol present in the surfactant comes from circulating lipoproteins, with very low density lipoproteins (VLDL) being considered the main vehicle for this distribution (15–17). Non-HDL cholesterol, including residual particles containing very low density lipoproteins (VLDL) and medium density particles, and TC/HDL-C were both indirect estimates of LDL-C particle number (18). Serum triglyceride and cholestonic acid levels were significantly increased in PAP patients (11, 17, 19). In addition, it has been suggested that serum lipid levels, particularly LDL-C and LDL-C/HDL-C levels, which correlate with PaO2 levels could reflect the severity of the disease in patients with PAP (20). Our analysis results showed that LDL-C has no significant difference between the control group and PAP patients, but there was significant difference between the mild-moderate PAP group and the severe PAP group. Consistent with reports in the literature, LDL-C/HDL-C was related to the severity of PAP. So far, an analysis of the lipid composition has shown that free cholesterol and cholesterol esters are greatly increased in PAP patients (21). However, the previous results were not supported by a large sample size, and the data on cholesterol and triglycerides were lacking. Recently, multiple studies have focused on the lipid ratios such as TC/HDL-C and TG/HDL-C in cardiovascular disease, metabolic disease, and cancer (22, 23). In the meantime, lipid ratios are useful to determine the severity of a non-alcoholic fatty liver disease (NAFLD) and coronary artery lesions (24, 25). However, few studies have focused on the association between lipid ratios and the severity of PAP. In our study, we found that patients with PAP had higher lipid ratios, including TC/HDL-C, TG/HDL-C, non-HDL-C, than the healthy subjects. Meanwhile, Patients with the severe PAP had higher TC/HDL-C and non-HDL-C than patients with the mild- moderate PAP. In addition, TC/HDL-C and non-HDL-C were independent risk factors for the occurrence and severity of PAP even after adjustment for other risk factors. Thus, we speculated that TC/HDL-C and non-HDL-C might participated in the occurrence and development of PAP. We will conduct basic research on the pathogenesis of PAP, which starting from the high level of blood lipids as the cause of increasing the burden on macrophages. Non-HDL-C, including VLDL remnant particles and intermediate density particles, and TC/HDL-C were both indirect estimate of LDL-C particle number (18). VLDL are mainly derived by the cholesterol-poor small LDL. Therefore, we also speculated that VLDL may be responsible for the association between TC/HDL-C, non-HDL cholesterol, and PAP, but the potential molecular mechanisms require further study.

Granulocyte–macrophage colony-stimulating factor (GM-CSF) plays an important role in the pathogenesis of autoimmune PAP. Some prospective multicenter studies of inhaled GM-CSF treatment have shown improvements in gas exchange, particularly in patients with severe PAP (26, 27). At the same time, Papiris showed that inhaled recombinant GM-CSF be used for 1 year or longer or increased the frequency to achieve both laboratory and clinical effectiveness (28). It is not clear why extending the use time or increasing the frequency of GM-CSF inhalation will make the effect more obvious. In another mechanism study, it was postulated that GM-CSF reversibly stimulates cholesterol clearance in macrophage and is concentration dependent. The loss of stimulation by GM-CSF changed the composition of the pulmonary surfactant by increasing the relative cholesterol level (29). Treatment with oral statins is associated with clinical, physiological and radiological improvement in autoimmune PAP patients, and *ex vivo* treatment with statins reduces cholesterol accumulation in alveolar macrophages (30). In light of this theory, we think that it is very important to find suitable lipid metabolism indicators to assess the severity of PAP and the effect of treatment.

ROC analysis results showed that TC/HDL-C, non-HDL-C, and TG (the AUROC >0.70) were acceptable predictors for the severity of PAP, but the AUROCs for non-HDL-C and TG were lower than TC/HDL-C. Thus, TC/HDL-C may be a potential surrogate for the severity of PAP.

Several limitations should be considered when interpreting the results of this study. First, the cross-sectional study design limits the ability to infer causality between lipid ratios and PAP. A prospective and comprehensive study to validate the role of circulating lipid ratios in PAP would be required. Second, we performed anti-GM-CSF autoantibody tests on all 122 patients and confirmed that only 6 cases were non-autoimmune PAP. We analyzed the data together which may lead to partial data bias. Finally, all participants were recruited from Nanjing, Jiangsu province, China. Therefore, it is uncertain whether these results are generalizable to other ethnic groups.

In conclusion, our study shows evidence that lipid ratios, especially TC/HDL-C are associated with the occurrence and severity of PAP. This study shows the promising values of TC/HDL-C is accessible biomarkers for the severity of PAP. Considering the potential importance of TG/HDL-C on the severity of PAP, it is important to check patients whose TC/HDL-C are elevated, especially TC/HDL-C over 5.05, lifestyles modification and stain therapy were needed for preventing the disease progression.

## Data Availability Statement

The original contributions presented in the study are included in the article/supplementary material, further inquiries can be directed to the corresponding author/s.

## Author Contributions

XY: writing—original draft preparation. YG: writing—reviewing and editing. QZ: formal analysis and validation. XQ and MT: data curation and validation. JD: provision of study patients and supervision. YZ: conceptualization and supervision. All authors read and approved the final manuscript.

## Conflict of Interest

The authors declare that the research was conducted in the absence of any commercial or financial relationships that could be construed as a potential conflict of interest.
